# Targeted Chromosomal Rearrangements via Combinatorial Use of CRISPR/Cas9 and Cre/*LoxP* Technologies in *Caenorhabditis elegans*

**DOI:** 10.1534/g3.118.200473

**Published:** 2018-06-27

**Authors:** Xiangyang Chen, Shimiao Liao, Xinya Huang, Ting Xu, Xuezhu Feng, Shouhong Guang

**Affiliations:** * Hefei National Laboratory for Physical Sciences at the Microscale, School of Life Sciences, University of Science and Technology of China, Hefei, Anhui 230027, P.R. China; † CAS Center for Excellence in Molecular Cell Science, Chinese Academy of Sciences, P.R. China

**Keywords:** pairing center, chromosomal inversion, CRISPR/Cas9, Cre/*LoxP*, balancer

## Abstract

Rearranged chromosomes have been applied to construct genetic balancers to manipulate essential genes in *C. elegans*. Although much effort has been put into constructing balancer chromosomes, approximately 6% (map units) of the *C. elegans* genome has not been covered, and this area lies mostly in pairing centers (PCs). Here, we developed a method for conditional chromosomal engineering through combinatorial use of the CRISPR/Cas9 and Cre/*LoxP* technologies. Functional DNA fragments containing *LoxP* sequences were inserted into designated genomic loci using a modified counterselection (cs)-CRISPR method. Then, heat-shock-induced Cre recombinase induced an inversion of the chromosomal region between the two *LoxP* sites. The chromosomal inversions were subsequently detected by the appearance of pharyngeal GFP. Through this method, we have successfully generated several chromosomal inversion lines, providing valuable resources for studying essential genes in pairing centers.

Chromosomal variations, including inversions, translocations and duplications, have been widely used as balancer chromosomes to maintain loss-of-function alleles of essential genes in a heterozygous form in *C. elegans* (Edgley *et al.* 2006). These structural abnormalities, which mostly arose from a number of random mutagenesis experiments, could reshape the recombination capabilities of the mutated chromosomes and lead to crossover suppression within specific regions (Rosenbluth and Baillie 1981; Zetka and Rose 1992; Zheng *et al.* 1999; Chen *et al.* 2015). Recent progress in targeted genome editing technologies has added to the repository of chromosome rearrangements (Choi and Meyerson 2014; Torres *et al.* 2014; Chen *et al.* 2015; Iwata *et al.* 2016; Dejima *et al.* 2018). At present, the existing balancer chromosomes cover a total of approximately 94% (map units) of the *C. elegans* genome. The remaining 6% (map units) is mostly located in the pairing center (PC) regions of LG II, LG IV and LG X (Figure S1) (Edgley *et al.* 2006; Chen *et al.* 2015; Iwata *et al.* 2016; Dejima *et al.* 2018). These pairing centers bind ZIM/HIM proteins to mediate homologous pairing and synapsis during meiosis in *C. elegans* (Rog and Dernburg 2013).

The new genome editing technologies, especially the CRISPR/Cas9 system, provide an excellent platform for efficient, site-specific chromosome engineering in a variety of organisms (Choi and Meyerson 2014; Chen *et al.* 2015; Iwata *et al.* 2016; Dejima *et al.* 2018). The Cas9 nuclease is guided by small guide RNA (sgRNA) to cleave specific DNA double strands and induce sequence mutations (Hsu *et al.* 2014). This technology has been used to induce mutations and construct transgenes in *C. elegans* (Cho *et al.* 2013; Dickinson *et al.* 2013; Friedland *et al.* 2013; Katic and Grosshans 2013; Tzur *et al.* 2013; Waaijers *et al.* 2013; Arribere *et al.* 2014; Kim *et al.* 2014; Liu *et al.* 2014; Paix *et al.* 2014; Zhao *et al.* 2014; Farboud and Meyer 2015; Norris *et al.* 2015; Paix *et al.* 2015; Ward 2015; Bell *et al.* 2016; Dickinson and Goldstein 2016; Paix *et al.* 2016; Schwartz and Jorgensen 2016). Meanwhile, by applying multiple sgRNAs, chromosomes can be engineered to generate site-specific rearrangements, including interchromosomal translocations and intrachromosomal inversions covering nonpairing center regions in *C. elegans* (Chen *et al.* 2015; Iwata *et al.* 2016; Dejima *et al.* 2018). However, it is still very challenging to successfully induce chromosomal rearrangements within pairing centers using the multiple-sgRNA strategy.

The Cre/*LoxP* technology has been utilized to induce chromosomal rearrangements for years (Yu and Bradley 2001). The site-specific Cre recombinase recognizes the *loxp* site and catalyzes the recombination of two *LoxP* sites to induce the excision or inversion of the flanked DNA segment, depending on the orientations of the two elements (Chen *et al.* 2016). The *LoxP* elements are usually inserted into specific genomic loci through an inefficient homologous recombination between endogenous DNA and exogenously added DNA molecules when cells propagate (Bouabe and Okkenhaug 2013). This recombination-mediated editing method has been applied to induce sequence deletions or inversions to manipulate gene expression in *C. elegans* (Hubbard 2014).

Here, we developed a strategy to conditionally induce chromosomal inversions covering pairing centers through combinatorial use of the CRISPR/Cas9 and Cre/*LoxP* technologies in *C. elegans*. We modified the cs-CRISPR method to efficiently insert *LoxP* elements into designated genomic loci. A DNA fragment expressing a heat-shock-inducible Cre recombinase was inserted into the *ttTi5605* locus through the Mos1 system. A strain containing two *LoxP* elements and the Cre-expressing element was constructed and heat-shocked to induce the expression of the Cre recombinase. The expression of Cre induced the recombination between two *LoxP* elements to trigger chromosomal inversions and resulted in pharyngeal GFP expression in the progeny. Through this method, we have successfully produced a number of chromosomal inversions covering the pairing centers. Thus, our method provides an effective platform for manipulating chromosomes in complex regions in *C. elegans*.

## Materials and Methods

### Strains

Bristol strain N2 was used as the standard wild-type strain. All strains were incubated on nematode growth medium (NGM) plates seeded with OP50 at 20°, except where otherwise noted (Brenner 1974). The strains constructed in this study are listed in Table S1.

### Plasmid construction

#### The construction of the sgRNA-expressing plasmids:

We manually searched for target sequences consisting of (N)_20_GG near the desired mutation sites. The targeted sequences are listed in Table S2. The sgRNAs used for multiple-sgRNA experiments were constructed as previously described (Chen *et al.* 2014). The modified sgRNAs ^(F+E)^, with an extended Cas9 binding structure and the removal of a potential Pol III terminator by an A-U base pair flip, were used in cs-CRISPR experiments (Zhao *et al.* 2016). We replaced the *unc-119* targeting sequence in the pU6::*unc-119* sgRNA^(F+E)^ expression vector with the desired target sequence using overlap extension PCR (Zhao *et al.* 2016). The pU6::*unc-119* sgRNA^(F+E)^ vector was diluted to 2 ng/µl and PCR-amplified to generate linear products. The PCR products were digested by the *Dpn*I restriction enzyme and transformed into Trans10 Chemically Competent Cells (Transgene Biotech, Beijing). We used the PhantaTM Super-Fidelity DNA polymerase (Vazyme Biotech, Nanjing, China, Cat. No. P501-d1/d2/d3) in all PCRs. The primer sequences used for the construction of the sgRNA expression plasmids are listed in Table S3.

#### The construction of the Cre-expressing plasmid:

For the construction of the Cre-expressing plasmid, a 400 bp *hsp-16.41* promoter region was amplified from N2 genomic DNA. The coding region of Cre was inserted with an intron sequence and PCR-amplified from pDD104 (a gift from the Bob Goldstein Laboratory) (Table S4). The fragments were cloned into pCFJ151 via the Gibson assembly method. The transgene was further integrated into the *C. elegans* genome by *Mos1*-mediated single copy insertion (MosSCI) into the *ttTi5605* site on LG II (Frøkjaer-Jensen *et al.* 2008).

#### The construction of the negative-selection plasmid:

The *unc-1p*::*unc-1(e1598)*::*unc-1 3′UTR* sequence was divided into three fragments, which were amplified from N2 genomic DNA. Then, the AMP-ORI (from pCFJ151) and the above three linear DNA fragments were joined together by Gibson assembly to form the pSG261 plasmid. The PCR product amplified from the pCFJ151 plasmid was digested by the *Dpn*I restriction enzyme before the assembly reaction. The sequences of the primers used for the construction of this plasmid are listed in Table S4.

#### The construction of the plasmid expressing wild-type DPY-8 protein:

The *dpy-8p*::*dpy-8*::*dpy-8 3′UTR* sequence was divided into two fragments, which were amplified from N2 genomic DNA. Then, the AMP-ORI (from pCFJ151) and the above two linear DNA fragments were joined together by Gibson assembly to form the pSG273 plasmid. The PCR product amplified from the pCFJ151 plasmid was digested by the *Dpn*I restriction enzyme before the assembly reaction. The sequences of the primers used for the construction of this plasmid are listed in Table S4.

#### The construction of donor plasmids and repair plasmids:

A series of donor vectors for Cre-mediated chromosomal inversions were constructed. We inserted the *LoxP* sequence into the pCFJ90 (*myo-2p*::*mCherry*::*unc-54 3′UTR*) and pSG259 plasmids (*myo-2p*::*gfp*::*unc-54 3′UTR*) using overlap extension PCR to generate linear products. These linear fragments were digested by the *Dpn*I restriction enzyme and transformed into Trans10 Chemically Competent Cells to construct the pSG264 (*myo-2p*::*LoxP*::*mCherry*::*unc-54 3′UTR*) and pSG265 (*myo-2p*::*LoxP*::*gfp*::*unc-54 3′UTR*) plasmids, respectively, serving as the donor of functional *LoxP* fragments (Table S4). For the *hygromycin* donor, a 1.1 kb *rps-11* promoter region (from N2 genomic DNA), the *hyg*::*unc-54 3′UTR s*equence (from pCZGY2728, a gift from the Zhiping Wang Laboratory) and the *myo-2p*::*LoxP*::*mCherry*::*unc-54 3′UTR* sequence containing AMP-ORI (from pSG264) were amplified (Table S4). These fragments were joined together by Gibson assembly to form the *hygromycin* donor plasmid (pSG266).

For construction of the *mCherry* cassette repair plasmids with recombination arms, the AMP-ORI (from pCFJ151), the *myo-2*::*LoxP*::*mCherry*::*unc-54 3′UTR + rps-11p*::*Hyg*::*unc-54 3′UTR* sequence (from pSG266), left recombination arms and right recombination arms (from N2 genomic DNA) were amplified. These fragments were joined together by the Gibson assembly method to form the pSG267, pSG269 and pSG271 repair plasmids, targeting the left ends of LG II, LG IV and LG X, respectively (Table S4). For construction of the *gfp* cassette repair plasmids, the AMP-ORI (from pCFJ151), the *LoxP*::*gfp*::*unc-54 3′UTR* (from pSG265), the *rps-11p*::*Hyg*::*unc-54 3′UTR* sequence (from pSG266), left recombination arms and right recombination arms (from N2 genomic DNA) were amplified. These fragments were joined together by Gibson assembly to form the pSG268, pSG270 and pSG272 repair plasmids, targeting LG II, LG IV and LG X, respectively, outside of the pairing centers (Table S4).

The ClonExpress MultiS One Step Cloning Kit (Vazyme Biotech, Nanjing, China, Cat. No. C113-01/02) was used in all Gibson assembly reactions. The PCR products amplified from plasmids were all digested by the *Dpn*I restriction enzyme before the assembly reactions. The primer sequences used for the construction of these plasmids are listed in Table S4. The non-sgRNA plasmids are summarized in Table 1.

**Table 1:** Non-sgRNA plasmids generated in this study

Plasmid	Functional DNA fragments
pSG260	*hsp-16.41p*::*SV40_NLS*::*Cre*::*tbb-2 3′UTR*
pSG261	*unc-1p*::*unc-1(e1598)*::*unc-1 3′UTR*
pSG264	*myo-2p*::*LoxP*::*mCherry*::*unc-54 3′UTR*
pSG265	*myo-2p*::*LoxP*::*gfp*::*unc-54 3′UTR*
pSG266	*myo-2p*::*loxP*::*mCherry*::*unc-54 3′UTR +* *rps-11p*::*HygR*::*unc-54 3′UTR*
pSG267	LG II (-8.47 cM) loci recombination template
pSG268	LG II (-18.01 cM) loci recombination template
pSG269	LG IV (-1.65 cM) loci recombination template
pSG270	LG IV (-27.20 cM) loci recombination template
pSG271	LG X (-6.16 cM) loci recombination template
pSG272	LG X (-21.60 cM) loci recombination template
pSG273	*dpy-8p*::*dpy-8*::*dpy-8 3′ UTR*

### Microinjection

#### cs-CRISPR-mediated DNA insertion:

DNA mixtures were microinjected into the gonads of young adult *C. elegans*. We injected 20 ng/µl repair plasmids, 50 ng/µl of the Cas9 expression vector (pDD162); 35 ng/µl of the sgRNA^(F+E)^ #1-, 35 ng/µl of the sgRNA^(F+E)^ #2- and 35 ng/µl of the sgRNA^(F+E)^ #3-expressing vectors (as indicated in the figures); 15 ng/µl of the pSG261 vector (a coinjected *unc-1(e1598)* dominant marker); and 3 ng/µl of the pSG259 vector (a coinjection marker that expresses GFP in the pharynx). The injection mix is summarized in Table S5. After they recovered from the injection, 10 worms were placed onto individual 10 cm NGM plates, and these worms were grown at 25°. Four to five days after the injection, 1 ml of 5 mg/ml hygromycin solution was added into each plate, and the plates were gently rotated to let the liquid cover the entire plate surface and incubated for another 3 days. If there were not enough bacteria in the plates, the hygromycin solution was mixed with fresh OP50 bacteria before adding to the plates. Then, from each plate, eight wild-type L4 stage worms or gravid adults that could move smoothly and did not express GFP in the pharynx were transferred to individual NGM plates. Three days later, for the *mCherry* cassette insertions, fluorescence signals were checked among the progeny, and homozygous strains were saved. For the *gfp* cassette insertions, 300 µl of 5 mg/ml hygromycin solution was added to each plate to check the hygromycin resistance phenotype of the F3 progeny, and the homozygous strains were saved. These homozygous F2 worms were transferred to 20 µl lysis buffer (500 µg /ml Proteinase K, 100 mM NaCl, 50 mM Tris, and 20 mM EDTA) and further confirmed by PCR amplification with primer pairs outside of the sgRNA-targeted regions. The primer sequences used for the PCR amplifications are listed in Table S6.

#### Dual-sgRNA-mediated chromosomal inversions:

For the inversion on LG IV, we injected 50 ng/µl of the Cas9 expression vector (Addgene plasmid #46168), 50 ng/µl of the sgRNA #1- and 50 ng/µl of the sgRNA #2-expressing vectors, and 5 ng/µl of the pCFJ90 vector. For the inversion on LG X, we injected 50 ng/µl of the Cas9-expressing vector (Addgene plasmid #46168); 50 ng/µl of the sgRNA #1-, 50 ng/µl of the sgRNA #2- and 50 ng/µl of the sgRNA #3-expressing vectors; and 5 ng/µl of the pCFJ90 vector. The injection mixes are summarized in Table S5. After they recovered from the injection, four to five worms were placed onto individual NGM plates. Three days after the injection, F1 animals expressing mCherry were transferred to individual NGM plates and allowed to produce F2 progeny for two to three days. From an F2 plate with dumpy animals, six to eight dumpy animals were transferred to a new NGM plate to lay F3 progeny. Then, F2 and F3 animals were harvested and transferred to 50 µl lysis buffer (500 µg /ml Proteinase K, 100 mM NaCl, 50 mM Tris, and 20 mM EDTA) and screened by PCR with specific primer pairs. The primer sequences used for PCR screening are listed in Table S5.

#### Heat-shock-induced chromosomal inversions:

L2-stage animals were transferred onto NGM plates (15 animals per plate for SGH637 and SHG638, 20 animals per plate for SHG639). The plates were heat-shocked at 34° for 4 hr. After the heat shock, the plates were kept at 25° for another 6-7 days. Then, F1 animals expressing GFP in the pharynx were selected and removed to individual NGM plates. Three days later, the homozygous F2 animals without mCherry expression were picked and maintained.

#### Imaging:

Images were collected using Leica DM2500 and M165 FC microscopes.

#### Egg-hatching assay:

Egg-hatching assays were performed as previously described (Chen *et al.* 2015). Hermaphrodites were placed on NGM plates containing 6-10 mm diameter bacterial lawns and allowed to lay eggs for 3-4 hr, then the parental animals were removed, and the number of eggs was counted. Three days later, the number of animals on the plates was counted again.

### Data availability

The authors affirm that all data necessary for confirming the conclusions of this article are present in the article, figures, and tables. The strains generated in this work are available upon request. Supplemental material available at Figshare: https://doi.org/10.25387/g3.6701705.

## Results

### The CRISPR/Cas9 technology with multiple sgRNAs failed to induce chromosomal inversions within pairing centers

The existing balancer system of *C. elegans* consists of a series of strains carrying chromosomal rearrangements, which cover approximately 94% (map units) of the total genome. All remaining uncovered regions lie within the pairing centers, and the highest percentage of that is concentrated in the PC regions of LGs II, IV and X (Figure S1). A series of nematode strains with chromosomal variations were obtained with the latest CRISPR/Cas9 technology, yet variations in pairing centers could not be obtained successfully (Chen *et al.* 2015; Iwata *et al.* 2016; Dejima *et al.* 2018). Here, we tried to use the multiple-sgRNA strategy to construct mutants with chromosomal inversions covering PC regions but without success.

For example, two sgRNAs targeting the *dpy-9* gene (LG IV, 257384-259204) and the *rde-8* gene (LG IV, 7101375-7103261), respectively, were used to induce a target inversion on LG II (Figure S2A). Loss of function in *dpy-9* and *rde-8* lead to dumpy morphology and an RNAi-defective phenotype (Rde), respectively (Brenner 1974; Tsai *et al.* 2015). We designed several sgRNAs targeting these two genes, and the gene editing efficiency of each sgRNA was assessed via scoring the production of Dpy progeny and Rde progeny, respectively. *rde-8* mutants were screened via identification of animals that suppressed the *unc-15* RNAi-induced paralysis phenotype. Two sgRNAs with high editing efficiency (*dpy-9* sgRNA, 20%; *rde-8* sgRNA, 37.5%) were selected. We injected 130 worms and selected 366 F1 worms expressing GFP in the pharynx. Sixty-five F1 worms gave birth to dumpy F2 animals. These dumpy animals were genotyped using specific primer pairs to screen for strains with the designated inversions. However, we were not able to obtain any mutant strains with the targeted chromosomal inversions (Figure S2B).

Second, we tried to induce an inversion on LG X through the use of sgRNAs targeting the *nrde-3* (LG X, 372235-376900) and *dpy-3* loci (LG X, 7536417-7537792). Loss of function in *nrde-3* results in suppression of *lir-1* RNAi-induced lethality, which is called nuclear RNAi defective (Nrde) (Guang *et al.* 2008; Guang *et al.* 2010; Mao *et al.* 2015). One sgRNA targeting the *dpy-3* gene (*dpy-3* sgRNA, 35%) and two sgRNAs targeting the *nrde-3* gene (*nrde-3* sgRNA #1, 12%; *nrde-3* sgRNA #2; 15%) were screened via scoring the production of Dpy progeny and Nrde progeny, respectively. These sgRNAs were coinjected into the wild-type N2 strain (Figure S2C). However, among the 103 F1 animals that produced F2 dumpy animals, no mutants exhibited the designated chromosomal inversions (Figure S2D).

We did not include donor molecules acting as repair templates during these multiple-sgRNA-mediated editing experiments. However, chromosomal rearrangements can be induced without exogenous repair templates through the CRISPR/Cas9 technology. For example, we have previously obtained a series of nematode strains with chromosomal translocations via the CRISPR/Cas9 technology without the use of donor molecules (Chen *et al.* 2015). Meanwhile, chromosomal inversions can be generated in the absence of donor molecules as well (Iwata *et al.* 2016). Even in the presence of donor molecules, many ligations were unlikely to be generated via homologous repair (Iwata *et al.* 2016; Dejima *et al.* 2018).

Taken together, these results implied that the pairing centers may possess a mechanism to prohibit CRISPR/Cas9-technology-induced chromosomal inversions.

### Strategy for chromosomal inversions through combinatorial use of the CRISPR/Cas9 and Cre/LoxP technologies

**Figure 1:**
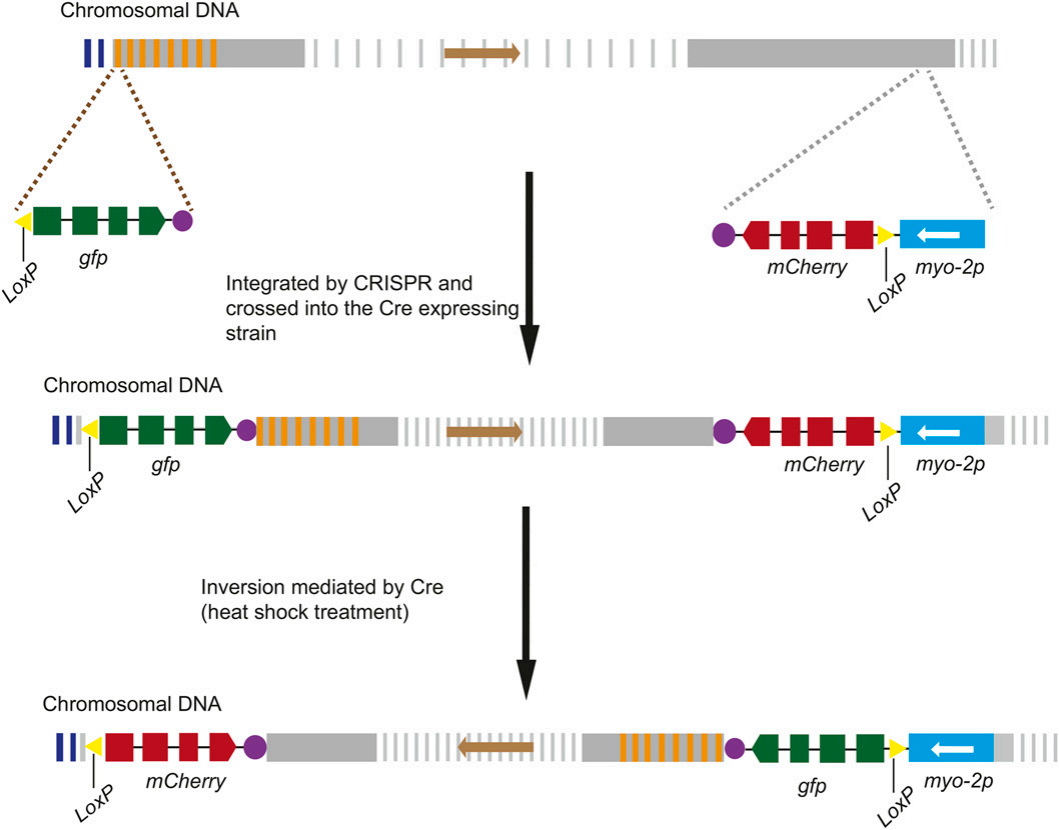
Strategy for chromosomal inversions through combinatorial use of the CRISPR/Cas9 and Cre/*LoxP* technologies. The *LoxP*::*gfp*::*unc-54 3′UTR* sequence was inserted at the end of the pairing center, and the *myo-2p*::*LoxP*::*mCherry*::*unc-54 3′UTR* sequence was inserted into the region outside the pairing center using CRISPR/Cas9 technology. These two transgenes were crossed together into a strain carrying an integrated *hsp-16.41p*::*SV40_NLS*::*Cre*::*tbb-2 3′UTR* sequence. Upon heat shock, the Cre recombinase was expressed and induced recombination between the two *LoxP* elements to generate a functional *myo-2p*::*LoxP*::*gfp*::*unc-54 3′UTR* sequence that led to the expression of pharyngeal GFP. The animals expressing GFP in the pharynx were selected as the targeted chromosomal variants. The white arrows and brown arrows indicate the orientations of the *myo-2* promoter and the chromosomal segments that lie between the loxP sites, respectively. The yellow triangles show the *LoxP* elements. The gray vertical lines and gray vertical rectangles indicate the chromosomal segments. The blue vertical lines and orange vertical lines indicate the telomeres and pairing centers, respectively (Rog and Dernburg 2013). The purple circle indicates the transcriptional termination site of *unc-54*.

To construct balancer chromosomes covering the pairing centers, we designed a heat-shock-induced inversion strategy using the Cre/*LoxP* technology in *C. elegans*. The schematic protocol is described in Figure 1. First, we constructed two plasmids, *LoxP*::*gfp*::*unc-54 3′UTR* and *myo-2p*::*LoxP*::*mCherry*::*unc-54 3′UTR*. Since the *LoxP*::*gfp*::*unc-54 3′UTR* does not contain a functional promoter, GFP will not be expressed. The *myo-2p*::*LoxP*::*mCherry*::*unc-54 3′UTR* plasmid contains the *myo-2p* promoter and can direct mCherry expression in pharyngeal muscle cells. Then, we used CRISPR/Cas9 technology to insert *LoxP*::*gfp*::*unc-54 3′UTR* into the left end of one chromosome and *myo-2p*::*LoxP*::*mCherry*::*unc-54 3′UTR* into a genomic locus outside the pairing center, respectively. Third, the strains carrying *LoxP*::*gfp*::*unc-54 3′UTR* and *myo-2p*::*LoxP*::*mCherry*::*unc-54 3′UTR* were crossed together into the strain carrying an integrated *hsp-16.41p*::*Cre*::*tbb-2 3′UTR* sequence. Upon heat shock, the expression of Cre recombinase was induced, which induced recombination between the two *LoxP* elements and generated a functional *myo-2p*::*LoxP*::*gfp*::*unc-54 3′UTR* sequence. Thus, the chromosomal rearrangement led to the expression of pharyngeal GFP, which became the selectable marker for the targeted chromosomal variants. In this protocol, the laborious microinjection and screening operations were replaced with the heat-shock treatment. Thus, a large number of animals could be manipulated simultaneously, increasing the probability of the identification of animals with the targeted rearrangements.

### Developing a modified cs-CRISPR method to insert LoxP elements into targeted genomic loci

The traditional method to insert *LoxP* elements into specific genomic loci relies on random recombination events between exogenous repair templates and endogenous DNA sequences. Here, we used modified cs-CRISPR/Cas9 technology to insert *LoxP* elements into designated genomic loci. To increase the efficiency of insertion, multiple sgRNAs targeting the same loci were used to induce DSBs. Because the pairing centers contain highly repetitive sequences, we inserted the DNA fragments carrying the *LoxP* elements into the nonrepetitive regions within the pairing centers using the CRISPR/Cas9 technology to avoid nontarget chromosome rearrangements.

**Figure 2:**
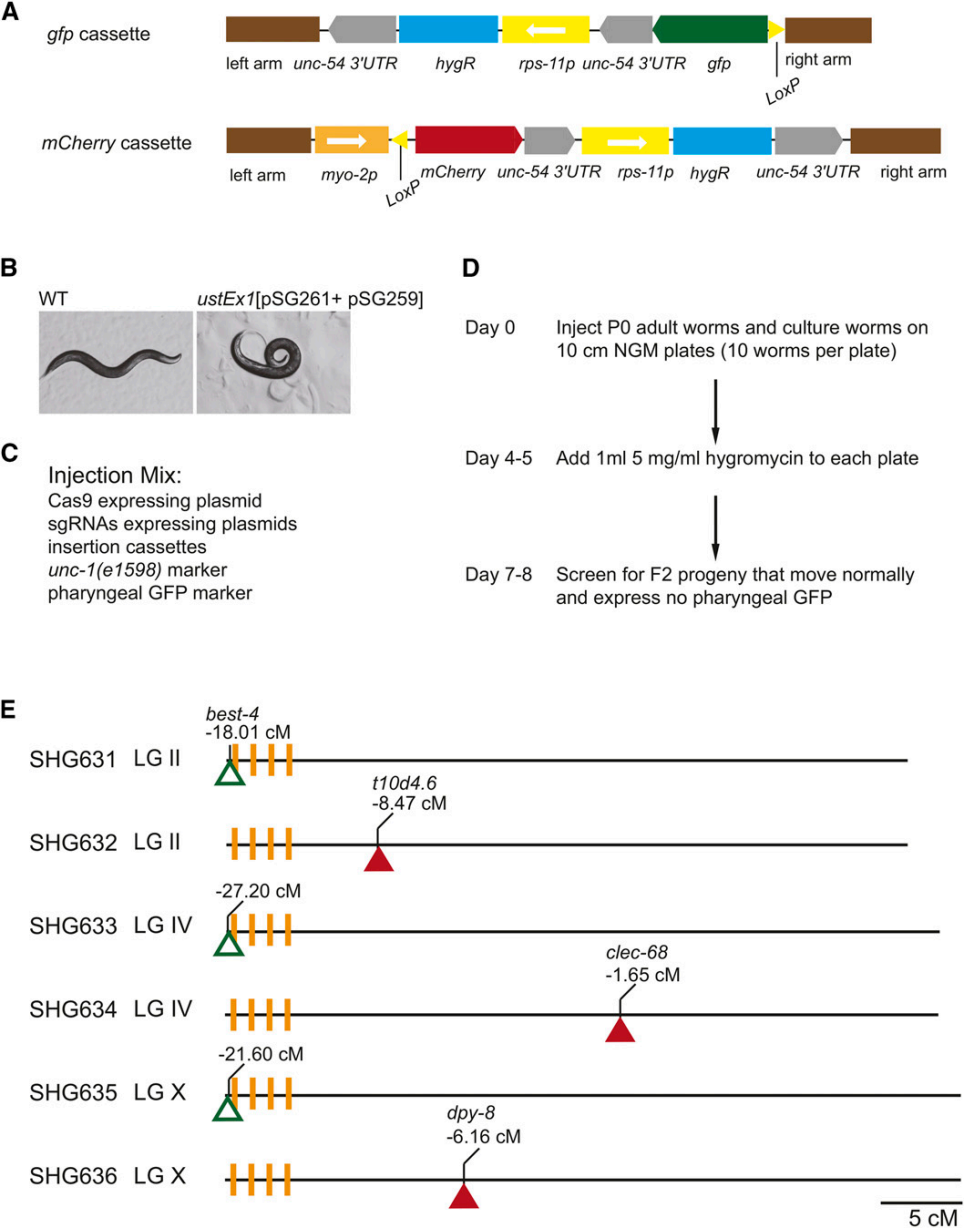
Targeted insertion of *LoxP* elements into genomic loci using CRISPR/Cas9 technology. (A) The hygromycin resistance gene was fused with the *LoxP*::*gfp*::*unc-54 3′UTR* sequence and *myo-2p*::*LoxP*::*mCherry*::*unc-54 3′UTR* sequence to construct the *gfp* and *mCherry* cassettes, respectively. The cassettes were fused with two recombination arms to construct repair plasmids. (B) Animals carrying the pSG261 plasmid (*unc-1p*::*unc-1(e1598)*::*unc-1 3′UTR*) exhibited a strong moving defect. (C) Injection mix for the insertions of the cassettes. (D) Schematic of the CRISPR/Cas9-mediated insertions. Plasmids expressing Cas9, multiple sgRNAs, the repair plasmids, and the negative selection markers were injected into the germlines of gravid adults. After they recovered from the injection, 10 worms were placed onto individual 10 cm NGM plates, and these worms were grown at 25°C. Four to five days later, 1 ml of 5 mg/ml hygromycin solution was added to each plate, and the plates were incubated at 25°C for another 3 days. Then, from each plate, eight wild-type L4 stage worms or gravid adults that moved smoothly and did not express GFP in the pharynx were isolated and transferred onto individual NGM plates. These selected strains were further confirmed through phenotypic analysis and PCR-based genotyping. (E) Schematic of strains with a *gfp* or *mCherry* cassette inserted into the designated genomic loci. The hollow triangles represent the *gfp* cassette, and the solid triangles represent the *mCherry* cassette. The orange vertical lines indicate the pairing centers.

To facilitate the identification of the target worms, the hygromycin resistance gene *(rps-11p*::*hypR*::*unc-54 3′UTR)*, serving as a positive selection marker, was inserted into the genome together with the *LoxP* elements (Figure 2A). We constructed a *gfp* cassette to combine the *LoxP*::*gfp*::*unc-54 3′UTR* sequence and the hygromycin resistance gene (Figure 2A). The *myo-2p*::*LoxP*::*mCherry*::*unc-54 3′UTR* sequence and the hygromycin resistance gene were fused together to generate the *mCherry* cassette. Meanwhile, we applied two negative selection markers, the *unc-1(e1598)* dominant marker (pSG261) and the pharyngeal fluorescence marker (pSG259) plasmid, to provide negative selection against extrachromosomal arrays. The *unc-1(e1598)* mutation leads to a dominant Unc phenotype (Park and Horvitz 1986). Worms carrying the pSG261 plasmid exhibited a strong moving defect (Figure 2B). We identified these worms and injected plasmids expressing Cas9, multiple sgRNAs, the repair plasmids, and the negative selection markers into the germlines of gravid adults (Figures 2C and 2D). After they recovered, the injected worms were transferred to new 10 cm NGM plates with 10 worms per plate. After hygromycin selection, eight L4 or young adult F2 worms that moved normally and did not express pharyngeal GFP were picked from each plate and transferred to individual NGM plates. The homozygous F2 worms were identified by phenotypic analyses and further confirmed by PCR-based genotyping. Through this strategy, we successfully inserted *gfp* cassettes into the left ends of LG II (-18.01 cM), LG IV (-27.20 cM) and LG X (-21.60 cM) (Figure 2E and Figure S3). The *mCherry* cassettes were inserted into LG II (-8.47 cM), LG IV (-1.65 cM) and LG X (-6.16 cM) outside of the pairing centers (Figure 2E and Figure S3).

The combination of multiple sgRNAs and the counterselection method provides a labor-saving strategy for the identification of nematode strains with exogenous DNA sequences inserted into the designated genomic loci (Figure S4). For the LG II (-18.01 cM) and LG II (-8.47 cM) cassette insertions, we injected 20 animals for each locus, and integrated lines were obtained on every plate (10 injected P0 animals per plate). For the LG IV (-27.20 cM and -1.65 cM) cassette insertions, 30 animals for each locus were injected, and 2 and 3 integrated lines were obtained, respectively. For the LG X (-21.60 cM and -6.16 cM) cassette insertions, 30 animals for each locus were injected, and 2 and 3 integrated lines were obtained, respectively.

### Heat-shock-induced chromosomal inversions

**Figure 3:**
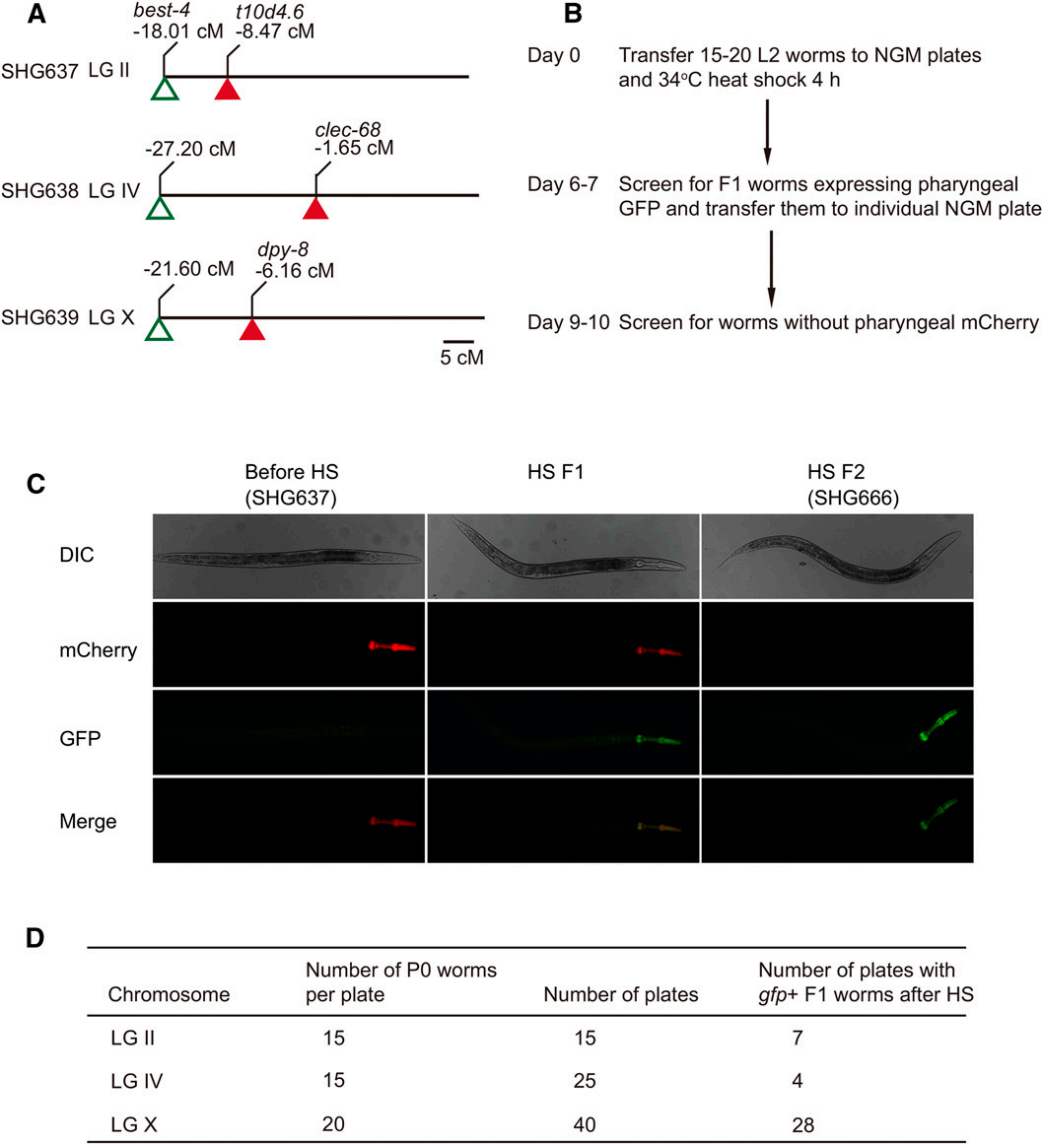
Heat-shock-induced chromosomal inversions. (A) The schematic of the strains containing the *gfp* and *mCherry* cassettes on the same chromosome, together with the Cre-expressing transgene. (B) The schematic of the heat-shock-induced chromosomal inversions. L2-stage animals were transferred onto NGM plates with 15-20 worms per plate. The plates were heat-shocked at 34°C for 4 hr. After heat shock, the plates were kept at 25°C for another 6-7 days. Then, F1 animals expressing GFP in the pharynx were isolated and transferred to individual NGM plates. Three days later, homozygous F2 animals without mCherry expression were picked and maintained. (C) Strategy for the identification of chromosomal variants on LGII. The parental worms expressed only pharyngeal mCherry. After heat shock, the targeted F1 heterozygous worms expressed both GFP and mCherry in the pharynx. The F1 worms could produce *gfp*-homozygous F2s that expressed only pharyngeal GFP. (D) Summary of heat-shock-induced chromosome rearrangements.

First, we constructed a strain carrying an integrated *hsp-16.41p*::*SV40_NLS*::*Cre*::*tbb-2 3′UTR* transgene to express Cre recombinase under a heat-shock promoter. The transgene was inserted into the *ttTi5605* locus (on LG II) using the MosSCI technology (Frøkjaer-Jensen *et al.* 2008). Then, we generated strains containing the *gfp* and *mCherry* cassettes on the same chromosome, together with the Cre-expressing transgene (Figure 3A). For LG II and LG IV, we sequentially crossed different *gfp* and *mCherry* cassettes on the same chromosomes into Cre-expressing animals to generate the SHG637 and SHG638 strains. For LG X, we directly injected the Cre-expressing animals with the *gfp* and *mCherry* cassettes. Then, the two strains were crossed together to generate the SHG639 strain.

To induce chromosomal inversions, we selected L2-stage worms for heat shock. We transferred 225 L2-stage SHG637 animals to 15 plates with 15 worms per plate. After heat shock, the animals were grown at 25° for another 6-7 days (Figure 3B). Among the 15 plates, we identified 7 plates with F1 worms expressing pharyngeal GFP (Figures 3C and 3D). The worms from different plates were saved as independent lines with chromosomal inversions. Similarly, the L2-stage worms of the SHG638 and SHG639 strains were heat-shocked to induce chromosomal inversions on LG IV and LG X, respectively. For SHG638 worms, among the 25 plates, we identified 4 plates with F1 worms expressing pharyngeal GFP (Figure 3D). For SHG639 worms, we transferred 800 L2-stage worms to 40 plates with 20 animals per plate. Among the 40 plates, we identified 28 plates with F1 worms expressing pharyngeal GFP (Figure 3D).

Most of the target F1 worms expressed both GFP and mCherry in the pharynx and were *gfp*-heterozygous. The *gfp*-homozygous F1 worms, which did not express pharyngeal mCherry, could be identified in a small portion of the plates through long-term culture. From each positive plate, four F1 animals expressing both GFP and mCherry in the pharynx were isolated and transferred to new NGM plates. Three days later, the homozygous F2 animals without mCherry expression were selected and maintained (Figures 3B and 3C). The worms isolated from different plates were saved as independent lines with targeted chromosomal inversions.

Interestingly, we heat-shocked young adult worms but failed to obtain F1 animals expressing pharyngeal GFP. We transferred 400 young adults to 40 plates with 10 animals per plate followed by 34° heat shock for 4 h. After heat shock, the animals were grown at 25° for another 4-5 days. However, among the F1 progeny on the 40 plates, we did not observe any worms expressing GFP in the pharynx. We speculated that the chromosomes of adult animals may adopt special structures that hinder Cre/*LoxP*-mediated chromosomal inversions. Further investigations are required to pinpoint such development-regulated alterations of chromosome structure.

### Chromosomal inversions suppressed recombination within the inverted regions

Through the Cre/*LoxP* strategy, we successfully generated chromosomal inversions covering the pairing centers of LG II, IV and X, which were named as *ustIn1*, *ustIn2* and *ustIn3*, respectively. To verify whether crossover processes were suppressed within the inverted genomic regions, we examined the recombination capabilities of different chromosomal regions in these mutants by crossing them to CB4856 animals. The CB4856 and N2 strains carry one single nucleotide polymorphism for roughly every 1,000 base pairs, which have been used as sequence markers for genotyping and SNP-based genetic mapping (Wicks *et al.* 2001).

We selected 3 SNPs on each chromosome for the linkage analysis, of which one was located near the right end of the chromosome and the other two were located near the inverted regions (Figure S5). After crossing the inverted strains with CB4856, the F2 progeny with GFP expression in the pharynx were transferred to individual NGM plates. The F2 *gfp*-homozygous animals were selected by visualizing fluorescence signals in the F3 generation. The F2 *gfp*-homozygous animals were picked and subjected to single-worm genotyping using PCR amplification of the SNP loci (Table 2 and Table S7). For *ustIn1*, among 178 homozygous F2 animals, there were 128 (72%) animals carrying the *WBVar02075879* variation, yet no worms carrying the *WBVar00162868* and *WBVar00168595* variations. For *ustIn2*, among 153 F2 homozygous animals, there were 125 (82%) animals carrying the *WBVar00199552* variation, yet no worms carrying the *WBVar0018281*6 and *WBVar00187093* variations. For *ustIn3*, among 183 homozygous F2 animals, there were 132 (72%) animals carrying the *WBVar02091917* variation but no worms carrying the *WBVar01978866* and *WBVar00077997* variations. These results demonstrated that recombination was suppressed within the inverted chromosomal regions.

**Table 2:** Recombination capability of different chromosomal regions of *ustIn1*, *ustIn2* and *ustIn3*

Strain	SNP Variation	Genomic location (cM)	Percentages of *gfp* homologous F2s with Hawaii variation (%) (total *gfp* homologous F2s)*
SHG637 (*ustIn1)*	*WBVar00162868*	LG II: -15.89	0 (178)
SHG637 (*ustIn1)*	*WBVar00168595*	LG II: -8.46	0 (178)
SHG637 (*ustIn1)*	*WBVar02075879*	LG II: 22.97	72 (178)
SHG638 (*ustIn2)*	*WBVar00182816*	LG IV: -26.69	0 (153)
SHG638 (*ustIn2)*	*WBVar00187093*	LG IV: -4.61	0 (153)
SHG638 (*ustIn2)*	*WBVar00199552*	LG IV: 15.28	82 (153)
SHG639 (*ustIn3)*	*WBVar01978866*	LG X: -21.41	0 (183)
SHG639 (*ustIn3)*	*WBVar00077997*	LG X: -7.40	0 (183)
SHG639 (*ustIn3)*	*WBVar02091917*	LG X: 24.09	72 (183)

* Chromosomal variants were crossed with CB4856 and the F2 progeny with pharyngeal GFP were transferred to individual NGM plates. The *gfp* homologous F2 animals were further isolated and single-worm genotyped.

Many of the balancer lines generated by chromosomal translocations suffer from aneuploidy, which is inconvenient for phenotypic analyses (Edgley *et al.* 2006). In contrast, inversion balancer lines have structural variations within their own chromosomes, are free from aneuploidy and are therefore easier to use (Zetka and Rose 1992). To examine whether our inversion lines were free from aneuploidy, we investigated the suppression of crossovers using egg-hatching assays of the *+/ustIn1*, *+/ustIn2* and *+/ustIn3* heterozygous strains (Table 3). The egg-survival rates of the *+/ustIn1*, *+/ustIn2* and *+/ustIn3* heterozygotes were 99.5% (n = 1989), 98.7 (n = 2321) and 99.2% (n = 2528), respectively, which were very close to that of wild-type animals. The total numbers of adult progeny per hermaphrodite of *+/ustIn1*, *+/ustIn2* and *+/ustIn3* heterozygotes were 301 (n = 10), 290 (n = 10) and 288 (n = 10), respectively, which were similar to that of wild-type animals as well (Table 3). These results suggested that there are no aneuploidies in the heterozygotes and that no crossovers occurred within the inverted regions.

**Table 3:** Egg-hatching assay of animals with inverted chromosomes

Genotype of parental hermaphrodite	Percentage of eggs reaching adulthood (%)*	Mean no. of adult progeny per hermaphrodite
*+/+*	99.6 (1390)	302 (10)
*ustIn1*	99.1 (1470)	279 (10)
*ustIn2*	98.9 (2090)	283 (10)
*ustIn3*	83.2 (1128)	188 (10)
*+/ustIn1*	99.5 (1989)	301 (10)
*+/ustIn2*	98.7 (2321)	290 (10)
*+/ustIn3*	99.2 (2528)	288 (10)

* Determined as described by Chen *et al.* (2015). The total number of eggs counted is in parentheses.

**Figure 4:**
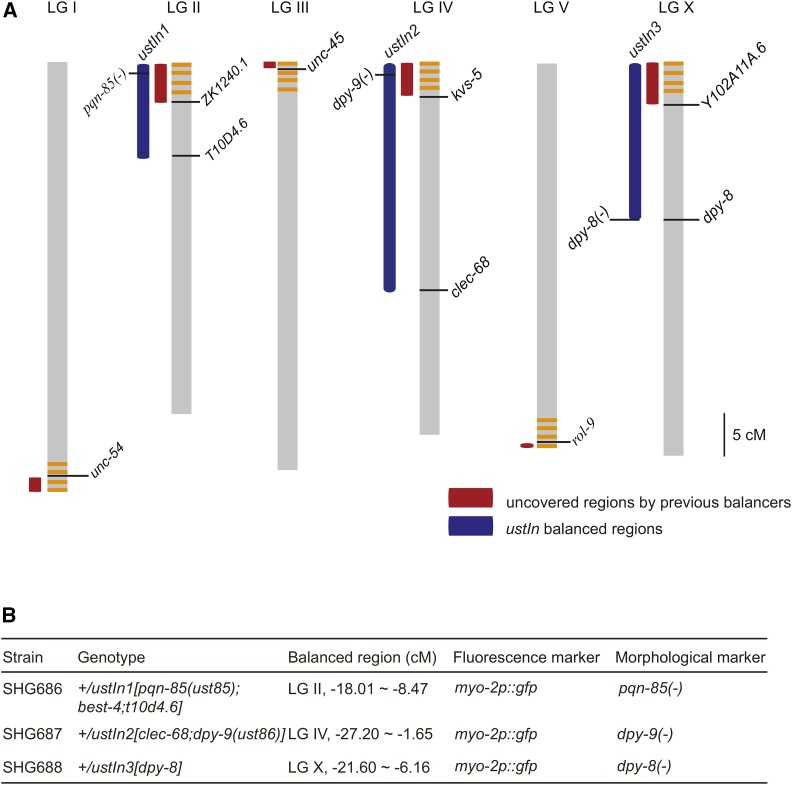
Summary of the chromosomal inversions generated in this study. (A) Schematic of the chromosomal inversions of *ustIn1*, *ustIn2* and *ustIn3*. The red bars indicate the regions not covered by previous balancer chromosomes. The blue bars indicate the regions balanced by *ustIn1*, *ustIn2* and *ustIn3*. The orange lines indicate the pairing centers. (B) Summary of the strains constructed with morphological markers. Loss-of-function mutations of *pqn-85* and *dpy-9* were introduced into *ustIn1* and *ustIn2*, respectively.

In summary, for *ustIn1*, recombination was suppressed from *t10d4.6* (LG II, -8.47 cM) to the left end of LG II but not suppressed from *t10d4.6* to the right end of LG II. For *ustIn2*, recombination was suppressed from *clec-68* (LG IV, -1.65 cM) to the left end of LG IV but not suppressed from *clec-68* to the right end of LG IV. For *ustIn3*, recombination was suppressed from *dpy-8* (LG X, -6.16 cM) to the left end of LG X but not suppressed from *dpy-8* to the right end of LG X (Figure 4A). To reduce the putative off-target mutations caused by genome manipulations, we outcrossed these strains with wild-type worms 4 times.

### The construction of balancer strains with chromosomal inversions

The strains generated in this work expressed GFP in the pharynx, making them appropriate tools for balancer construction. In addition, *ustIn3* is linked with a mutation in *dpy-8*, which can act as a visible marker as well. Morphological markers were also introduced into each inverted chromosome through knocking out *pqn-85* and *dpy-9* in *ustIn1* and *ustIn2*, respectively, using CRISPR/Cas9 technology (Figure 4A). PQN-85 is required for faithful chromosome segregation and embryonic viability (Seitan *et al.* 2006). Loss of function in *pqn-85* results in a sterile phenotype. Thus, the +/*ustIn1[pqn-85(ust85)]*, *+/ustIn2[dpy-9(ust86)]* and +/*ustIn3[dpy-8]* balancer strains can be used to balance and maintain loss-of-function alleles of essential genes within the inverted chromosomal regions (Figure 4B). Mutations in genes of interest can be introduced into the normal chromosomes of these balancers using the CRISPR/Cas9 technology, as previously described (Chen *et al.* 2015).

As the *dpy-8* animals were difficult to mate, we rescued the Dpy phenotype of the *ustIn3* strain with a PCR product expressing wild-type *dpy-8* and generated a strain (SHG759) carrying extrachromosomal (Ex) arrays of *dpy-8(+)* as previously described (Figure S6) (Dejima *et al.* 2018). We confirmed that *ustIn3* males carrying these extrachromosomal (Ex) arrays of *dpy-8(+)* could mate well (data not shown).

### The construction of a W07E6.2 balancer using the CRISPR/Cas9 system

**Figure 5:**
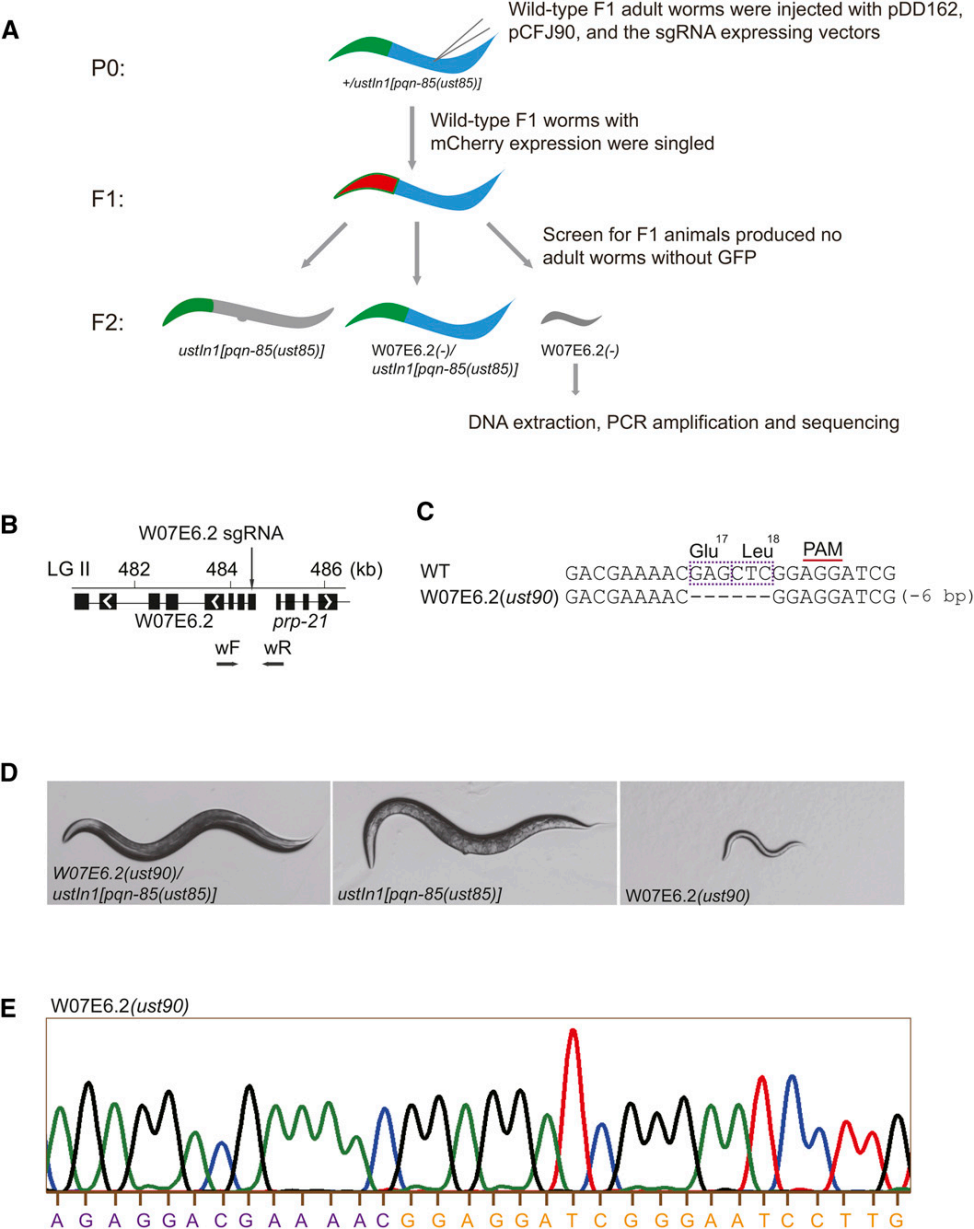
Construction of W07E6.2 balancer using the CRISPR/Cas9 system. (A) Schematic of gene editing and balancer construction for the W07E6.2 gene using CRISPR/Cas9 technology. The dominant transformation marker mCherry was coinjected with the Cas9 and sgRNA expression plasmids. The arrested larvae of F2 animals without pharyngeal GFP were selected and sequenced. (B) Schematic of the W07E6.2 gene. The position of the sgRNA-guided cleavage site and PCR primers for genotyping are indicated. (C) Sequence alignments of the W07E6.2 gene in wild-type and mutant animals. The PAM sequence is labeled and overlined. The dashed purple rectangles indicate the deleted open reading frames (ORFs). (D) Phenotypes of the progeny resulting from the self-fertilization of *W07E6.2(ust90)/ustIn1[pqn-85(ust85)]* heterozygotes. (E) Chromatogram of DNA sequencing of the W07E6.2 locus in animals without pharyngeal GFP after ten generations of *W07E6.2(ust90)/ustIn1[pqn-85(ust85)]* heterozygotes. Thirty animals were sequenced separately, and one of them is shown here.

We examined whether *ustIn1(II)* could balance a recessive lethal mutation within the inversion interval. The W07E6.2 gene, which is required for ribosome biogenesis, was selected to be knocked out in the SHG686 (+/*ustIn1[pqn-85(ust85)]*) animals. We injected a plasmid expressing sgRNAs targeting the first exon of W07E6.2, the pDD162 vector and the pCFJ90 vector (a coinjection marker that expressed mCherry fluorescent protein in the pharynx) into the germline of SHG686 animals (Figures 5A and 5B). Wild-type F1 animals with both pharyngeal mCherry and GFP were singled to individual NGM plates to lay F2 progeny. After 3-4 days, the phenotypes of the F2 animals without pharyngeal GFP were examined. Among 22 selected F1 animals, 1 F1 animal did not produce adult worms without pharyngeal GFP, and its progeny without pharyngeal GFP arrested at the L2 stage. These arrested worms were PCR-amplified and sequenced. The *ust90* mutation in W07E6.2 resulted in a deletion of two amino acids: Glu18 and Leu19 (Figure 5C). Thus, we obtained the balancer strain SHG753 (W07E6.2*(ust90)/ustIn1[pqn-85(ust85)]*).

We cultured this balancer strain for more than 10 generations to examine whether *ustIn1(II)* could stably balance the recessive lethal mutation in W07E6.2. SHG753 segregated three phenotypes: WT animals (W07E6.2*(ust90)/ustIn1[pqn-85(ust85)]* heterozygotes with pharyngeal GFP), lethal animals (W07E6.2*(ust90)* homozygotes without pharyngeal GFP), and sterile animals (*ustIn1[pqn-85(ust85)* homozygotes with pharyngeal GFP) (Figure 5D). The segregation of these phenotypes was maintained through more than 10 generations, suggesting that *ustIn1(II)* could stably suppress recombination in the covered genomic region. After 10 generations, we selected 30 worms without pharyngeal GFP and sequenced their W07E6.2 loci. All the worms exhibited homozygous W07E6.2*(ust90)* mutations (Figure 5E).

Overall, this balancer system is a useful tool for the maintenance of lethal mutations.

## Discussion

Our work provides a useful platform to combine the CRISPR/Cas9 technology and the Cre/*LoxP* system to generate nematode strains with designated chromosomal inversions covering complex chromosome regions. Through the method, we have constructed balancers covering the pairing centers of LG II, IV and X. Together with the classical balancers and crossover suppressors generated by latest genome editing technologies, the balancer system now covers more than 99% of the *C. elegans* genome, while leaving the right most end of LG I (0.2 Mb) and left most end of LG III (0.49 Mb) uncovered.

To efficiently insert the *LoxP* elements into specific genomic loci, we developed a modified cs-CRISPR method. The *HygR* gene, as a positive selection marker, was co-inserted into the genome together with functional *LoxP* elements. The *unc-1(e1598)* dominant marker served as a negative selection marker to eliminate worms carrying extra-chromosome arrays. Meanwhile, the co-injection of multiple sgRNAs promoted the formation of DSBs to induce the homology-directed DNA repair. Through this method, the transgenic animals with *LoxP* elements insertions can be easily identified by the injection of a few animals.

Our approach is also applicable to non-PC regions to induce chromosome inversions. However, given those non-PC regions can be easily engineered by CRISPR/Cas9 technology with multiple sgRNAs, it is unnecessary to include the *LoxP* method. The combination of CRISPR/Cas9 and Cre/*LoxP* systems is particularly useful for complex chromosome regions.

Many balancers are marked with the multi-copy GFP transgenes for the identification of larval-arrested worms with targeted mutations from the heterozygous balancer strains. Here, we introduced morphological markers, *dpy-8*, *dpy-9*, and *pqn-85*, into the inverted regions to simplify the balancer identification. In addition, the pharynx-expressed GFP was also linked to the inverted chromosomes. Including these markers will ease the future applications of the balancer strains.
